# The Geneva University *Global Health and Human Rights* Summer School: A 5-Year Intercultural Collaborative Experience

**DOI:** 10.3389/fpubh.2018.00128

**Published:** 2018-05-07

**Authors:** Philippe Chastonay, Emmanuel K. Mpinga

**Affiliations:** ^1^University of Fribourg, Fribourg, Switzerland; ^2^Institute of Global Health, University of Geneva, Geneva, Switzerland

**Keywords:** human rights, global health, training, summer-school, education

## Abstract

Education and training in human rights has been set as a priority by the United Nations. Health and human rights are closely related. Training professionals from various backgrounds in human rights might ultimately contribute to improve the health of individuals and communities. We present the 5 years’ experience with a 3-week residential *Global Health and Human Rights Course* developed at the University of Geneva and implemented with the support/participation of international organizations (IOs) and non-governmental organizations active in the health and human rights sector. Over the years, roughly 150 students from 43 nationalities, with many different educational backgrounds, attended the course. The male/female ratio was 1/5. The adopted educational approach was multifold and comprised lectures from academics and experts with field experience, group work, individual case studies, journal clubs, and site visits. Evaluation data show that site visits at IOs were highly appreciated as well as networking opportunities among students, with academics and experts with field experience. The variety of topics discussed was, at times, “too much”; yet, it allowed students to measure the extent of the challenges the field is facing. The adopted active learning approach facilitated the exchange of experiences among students and allowed them to get acquainted with different cultural sensitivities. The *Global Health and Human Rights Summer-School* of the University of Geneva allowed its participants, coming from all over the world, to identify challenges of the interlinked fields of health and human rights, reflect upon their underlying causes, and imagine possible solutions. Sharing our experience will hopefully help passionate educators around the world to develop similar programs.

## Introduction

Health and human rights are closely related (1, 2). Violating basic human rights can heavily impact in a negative way the health of individuals and communities, e.g., torture, domestic violence and stigmatization of persons with mental disorder and their families (3–5). Violating further basic human rights might also lead to inequality and discrimination in access to health-care services and/or to disease prevention programs (6). As a matter of fact, the concrete realization of the very importance of human rights in terms of health has crystallized in the context of the HIV/AIDS epidemics. Studies have shown as early as in the late eighties that the promotion and protection of human rights were essential in order to ensure an effective response to the HIV/AIDS epidemics (7–9).

Yet, when it comes to the relationship between health and human rights, there are several dominating schools of thought. The normative approach insists on developing legal and regulatory frameworks (10); the ethical approach puts emphasis on the value of social justice (11); the advocacy approach considers a priority the spreading of awareness of health and human rights topics among the population and professionals (12); finally, the interventionist approach proposes to use human rights in order to change traditions (13). Gruskin et al. take into account such theoretical pluralism when they state: “*We need to recognize the different ways in which health and human rights can be achieved. These ways can be categorized as advocacy, application of legal standards, and programming, including service delivery*” (14).

Training professionals from various backgrounds in human rights might ultimately contribute to improve the health of individuals and communities (15). The importance of such training programs has also been stressed by the *UNAIDS International Guidelines on HIV/AIDS and Human Rights* (16). There have been critical voices that have questioned whether Human Rights training programs actually are capable of bringing some change in the relationship between health and human rights, especially insisting on the necessity of different educational approaches (17). The UN Manual on Human Rights Training Methodology goes into the same direction (18): it gives extensive guidelines on how to implement effectively a training program on human rights insisting on specifically tailoring the courses objectives on the participants needs; it also stresses the necessity of promoting a participatory approach (problem-solving, group work, field visits) as well as an appropriate selection of trainers and resource persons (e.g., practitioners with field experience and academic experts in human rights). People trained in human rights may act as multipliers for human rights education and promotion as shown in various settings [non-governmental organizations (NGOs), schools, businesses, prisons, health-care services, etc.] (19–24).

Geneva is the “self-proclaimed” capital of human rights due to the presence of many international organizations (IOs) and NGOs committed to support and promote human rights (i.e., UN Human Rights Council, International Labor Organization ILO, International Committee of the Red Cross ICRC, World Health Organization WHO, World Intellectual Property Organization WIPO, etc.). As a matter of fact, a lot of *Health and Human Rights* expertise exists in Geneva thanks to the pioneering work of those organizations in the field of health and human rights. WHO has developed a conceptual framework linking health to human rights (25), based on the right to health of its Constitution and further developments of the concept (26), as well as specific guidelines, educational tools, and grass-root projects relying on human rights to promote health: let us mention as an example its work in supporting countries to develop human right-oriented mental health policies and plans (5). ILO has been the leading force worldwide in fighting child labor and its impact on the health of children through advocacy, educational programs and community projects (27). WIPO has been a major contributor to the Doha Declaration (intellectual property rights and public health), which has insured better access to medicines and health care to millions of patients since its adoption in 2001 (28).

This unique setting was put forward by the University of Geneva when the decision was taken to develop and implement several training programs related to human rights: e.g., a Certificate of Advanced Studies (CAS) in Human Rights was developed at the Law School (29); with the support of the Geneva Academic Society, the Federal Agency Against Racism and the Federal Agency for Cooperation and Development, a CAS in Health and Human Rights, as a residential program for local health professionals and social workers (30) and as a distance learning e-program targeting professionals from six French-speaking African (31) was implemented at the Medical School: a pre-graduate one-semester elective in Health and Human Rights was integrated into the pre-graduate medical curriculum (32).

In order to reach out to students from all over the world, the University of Geneva has launched a few years ago an ambitious summer school program, presently including courses in as many as 16 topics related to specific expertise among Geneva University researchers and their collaborative partnership with IOs located in Geneva (33). One of these courses is the 3-week residential *Global Health and Human Rights Course* of which we present hereafter the objectives, its educational approach, its collaborative perspective with IOs, its student body characteristics as well as some evaluation data of the past 5 years with the ambition that sharing our experience might be an incentive for passionate educators around the world to develop similar programs.

## The Program

The program was developed by the public health teaching staff of the Institute of Social and Preventive Medicine (presently Institute of Global Health) of the University of Geneva in close collaboration with experts from IOs and NGOs active in the field of Health and Human Rights and located in Geneva. The first version of the program was submitted for revision and approval to the Education Taskforce (comprising Human Rights experts) of the Coimbra Group, an association of 39 European Universities from 23 countries dedicated to promote academic collaboration, excellence in learning and research, and service to society (34). The two authors of the article represent the core faculty over the studied period. Tuition fee was CHF 2000 with some scholarships available from the University of Geneva.

### The Objectives

By the end of the course, the students should be able:

– to define the basic concepts of human rights and its links to global health and list the main issues of global health;
– to list the human rights instruments and tools of prime importance to health.

Furthermore, the students, by the end of the course, should be familiar with:

– the impact of globalization on the health of population;
– health and human rights challenges UN Agencies are confronted with;
– several public health issues where global health and human rights collide (e.g., violence against women, torture, discrimination against people living with a handicap, etc.);
– health policy fostering non-discrimination of patients and specific vulnerable groups.

Finally by the end of the training module, the students should be able:

– to integrate Global Health and Human Rights issues into professional practice;
– to lobby in favor of basic respect of human rights in the field of public and community health.

### The Target Population

Pre-graduate and post-graduate university students represent the target population with no restriction concerning the study-field background, nor the nationality. The yearly number of students was limited to 30. Admission criteria included a bachelor degree from a University or a University of Applied Sciences, some experience with health or human rights projects/issues, a short written essay on human rights challenges in their respective countries (not every year however). Furthermore, special attention was paid to having participants from high-, middle-, and low-income countries.

Over the years, the students mean age was 26 years. The male–female ratio was 1/5 (note quite surprising since it has been reported that within the health sector, in many countries women comprise over 75% of the workforce) (35). Countries of origin (*n*: 43) of the students included Australia, Austria, Belgium, Brazil, Cameroon, Canada, China, Columbia, Congo, Costa Rica, Cyprus, Czech Republic, Denmark, Egypt, Finland, France, Germany, Ghana, Greece, India, Indonesia, Italy, Kenya, Mexico, Netherlands, Nigeria, Norway, Peru, Poland, Serbia, Singapore, South Korea, Spain, Sweden, Switzerland, UK, Tanzania, Tunisia, Uganda, Ukraine, USA, and Zambia. Professional background or field of study of the participants included anthropology, biology, criminology, earth sciences, ethics, health policy, health promotion, international relations, law, medicine, nursing, philosophy, psychology, and public health.

### The Partner Institutions

Over the years, regular partner institutions have been IOs located in Geneva such as the WHO, the ILO, the International Committee of the Red Cross ICRC as well as NGOs such as the International AIDS Society, the NGO OIDEL focusing on the right to education, the NGO Waterlex specialized in legal and policy skills relating to the human rights to water and sanitation. These partner institutions opened the doors of their headquarters to students for visits, organized workshops on some specific health and human rights issues, developed case studies or offered staff expertise for lectures and discussion podiums.

### The Educational Approach

The educational approach was multifold, but heavily centered on active learning, inter-student exchange of experience and confrontation with professionals active in the field of health and human rights, notably through site visits.

– *Individual readings*: a syllabus of relevant documents was handed out to students for preparatory reading; some documents were used as basis for *Journal Clubs* during the program.
– *Lectures*: lectures on key issues related to “health and human rights” were given by Geneva academic staff, by experts from IO and NGOs located in Geneva as well as by teachers from various universities abroad. Examples of topics taught appear in Table 1. Over the years, different speakers illustrated the learning objectives from various perspectives.
– *Group work*: The program included several group works a week followed by in class presentations. Examples are given in Table 2.
– *Case studies*: Individual home work included at least one specific case study each student was expected to finalize. Examples are given in Table 3.
– *Site visits*: Site visits at IOs, mainly WHO, ILO, ICRC, and the UN-OHCHR, allowed the students to get acquainted with the organization, the projects and programs as well as the commitment of those organizations in supporting basic human rights in a health perspective. These were essentially full-day visits varying from year to year according to the Health and Human Rights priority projects of the moment of each institution. Participants of successive cohorts were able to become familiar with various human rights issues those institutions are confronted with, such as:
  – at WHO headquarters, examples of restrictions of basic human rights (association, free movement) related to the International Health Regulations;
  – at the ILO headquarters, specific examples of violations of basic human rights occurring at the workplace (harassment at work) and human rights tools/programs that can counteract such situations;
  – at the ICRC headquarters, examples of recent violations of the Geneva Conventions in various conflicts zones, notably also the growing amount of attacks against health-care workers, hospitals, and ambulances depriving people in war zones of life-saving treatments and ways ICRC and NGOs try to handle the situation.
  – At the UN-HCHR Office, the challenges and opportunities of the Human Rights education in the health sector.

**Table 1:** Examples of lectures on specific Health and Human Rights topics over the years.

Topic	Host institution of the lecturer
Human Rights and Public Health: History, principles, norms, and standards	University of South Wales
International Human Rights Treaties	University of Geneva
The Right to Health	University of Neuchâtel
Monitoring the Right to Health	UN Human Rights Office of the High Commissioner
Social and Economic Rights	UN Human Rights Office of the High Commissioner
Health and Human Rights Research Challenges	University of Geneva
Social Determinants of Health and Equity	World Health Organization (WHO)
Discrimination in Health Systems	Wichita State University
Access to Health Care	International Labor Organization
Mental Health in a Human Rights Perspective	University of Geneva
Reproductive Health and Human Rights	United Nation Population Fund
Drug Policies and Human Rights	University of Geneva
The Health of Women	University of Geneva
Access to Health Care in Detention	NGO Association for the Prevention of Torture
Health, Migration and Prostitution	University of Applied Sciences of Geneva
The Right to Health of People with Disabilities	WHO
AIDS and Human Rights	UNAIDS
The Right to Food and Health	Geneva Academy of International Humanitarian Law and Human Rights
Public Health Emergencies and Human Rights	MSF Médecins Sans Frontières
Ethics in Humanitarian Action	International Committee of the Red Cross
Water-Sanitation and Health	NGO Waterlex
Legal and Human Rights Aspects of the International Health regulation	WHO
Environment and Health Inequalities	University of Geneva
Human Rights Education for the Health Sector	International Organization for the Right to Education and Freedom of Education

**Table 2:** Examples of group work topics, assignments, and recommended basic readings.

Topic	Assignment: *prepare a 20-min presentation (followed by a 10-min discussion) taking into account the following points*	Recommended readings
The Right of Children living with disabilities	The importance of the problem in a public health perspective (e.g., prevalence, associated morbidity, costs, etc.) at national or international level. The link between the situation of children living with a disability and the violation of their basic human rights (e.g., invisibility of disabled children, discrimination at community and health system levels, lack of resources, etc.) The human rights tools (conventions, protocols, etc.) at disposal to challenge discrimination of disabled children, to promote the rights of disabled children	Promoting the rights of Children with disabilities http://www.un.org/esa/socdev/unyin/documents/children_disability_rights.pdf Convention against discrimination in education http://unesdoc.unesco.org/images/0011/001145/114583e.pdf#page=118 Convention of the rights of the child http://www.unicef.org/crc/index_30160.html Convention of the rights of persons with disabilities http://www.un.org/disabilities/convention/conventionfull.shtml World Report on disabilities http://whqlibdoc.who.int/publications/2011/9789240685215_eng.pdf
Living with HIV/AIDS: discrimination in the health system and in the community	The importance of the problem in public health perspective (e.g., prevalence, associated morbidity, costs, etc.) at national or international level. The link between people living with HIV/AIDS and the violation of their basic human rights (e.g., limited access to appropriate medication, discrimination at community and health system levels, lack of resources in research and in prevention, right of women to specific information, etc.) The human rights tools (conventions, protocol) to challenge discrimination of rights of people living with HIV/AIDS and to promote their rights, etc.	UNAIDS. HIV-Related Stigma, Discrimination and Human Rights Violations Case studies of successful programs http://data.unaids.org/publications/irc-pub06/jc999-humrightsviol_en.pdf Parker R, Aggelton P. HIV/AIDS-related Stigma and Discrimination: A Conceptual Framework and an Agenda for Action http://pdf.usaid.gov/pdf_docs/Pnacq832.pdf Mawar N., Sahay S., Pandit A. et al. The third phase of HIV pandemic: Social consequences of HIV/AIDS stigma & discrimination & future needs
Violence against women	The importance of the problem in a public health perspective (e.g., prevalence, associated morbidity, costs, etc.) at national or international level The link between women victims of violence and the violation of their basic human rights The human rights tools (conventions, protocols, etc.) at disposal to challenge discrimination of rights of women victims of violence to promote their rights	WHO Addressing violence against women and MDG 2005http://www.who.int/gender/documents/MDGs&VAWSept05.pdf UN. Declaration on the elimination of Violence against women http://www.unhchr.ch/huridocda/huridoca.nsf/(symbol)/a.res.48.104.en National Online Resource Centre on Violence against women http://www.vawnet.org/ OMCT. 370 articles on violence against women http://www.omct.org/violence-against-women/

**Table 3:** Example of a case study.

Situation	Assignment	Recommended reading
Excerpts from *Social Work & Society, Volume 11, Issue 1, 2013*: Testimonies of people (or family members living with people) suffering from mental disorders *“*… *we were a happy family but things changed when I got sick, my husband deserted me and went for another woman accusing me of being a witch* … *he took our two children with him and I hardly see them* … *I don’ t have a choice, do I?”* *“* … *my friends changed their attitudes toward me when they became aware of my son’s mental illness, some have stopped visiting me for fear of being attacked by my son.”* *“* … *when I came home from the psychiatric hospital, I went back to my workplace but my manager said I was sick and that he cannot afford to lose customers because of me* …*”* *“*… *I am a dressmaker but have stopped sewing* … *lost my customers after was diagnosed with mental illness* … *opened my shop a couple of times but nobody came around, even my apprentices refused to come to work.”*	Write an essay of maximum 10 type-written pages on the situation of people suffering from mental disorders and their families in your country based on available local and national data. Include if possible reported testimonies on discrimination and stigmatization of people (and their families) suffering from mental disorders. Provide information of national mental health policies	Prevention and Promotion in Mental Health http://www.who.int/mental_health/media/en/545.pdf Mental health Action Plan 2013–2020 http://www.who.int/mental_health/publications/action_plan/en/ Mental Health, Human Rights and Legislation http://www.who.int/mental_health/policy/legislation/Resource%20Book_Eng2_WEB_07%20%282%29.pdf Mental Health declaration of Human Rights http://www.cchr.org/about-us/mental-health-declaration-of-human-rights.html

The site visits also allowed students to better understand to what extend these IOs did crucial advocacy job at a global level as well as assumes a coordinating role for field projects in various countries/regions.

Table 4 shows which learning objectives were essentially acquired through which educational tool, though it must be underlined that there were no rigid boundaries; as a matter of fact various intermingled approaches allowed to achieve the different learning objectives. Variations over the years occurred, particularly in relation to the availability of the speakers, yet the learning objectives remained the same over the years.

**Table 4:** Achievement of learning objectives through various educational approaches.

Learning objectives	Main educational approach
To define the basic concepts of human rights and its links to global health and list the main issues of global health;	Individual readings, lectures
To list the human rights instruments and tools of prime importance to health	Individual readings, lectures
The impact of globalization on the health of population	Lectures, group work, journal clubs
Health and human rights challenges UN Agencies are confronted with	Site visits
Several public health issues where global health and human rights collide (e.g., violence against women, torture, discrimination against people living with a handicap, etc.)	Case studies, group work
Health policy fostering non-discrimination of patients and specific vulnerable groups	Lectures, group work, site visits
To integrate global health and human rights issues into professional practice	Group work, site visits
To lobby in favor of basic respect of human rights in the field of public and community health	Group work, site visits

## Evaluation Data

**Figure 1:**
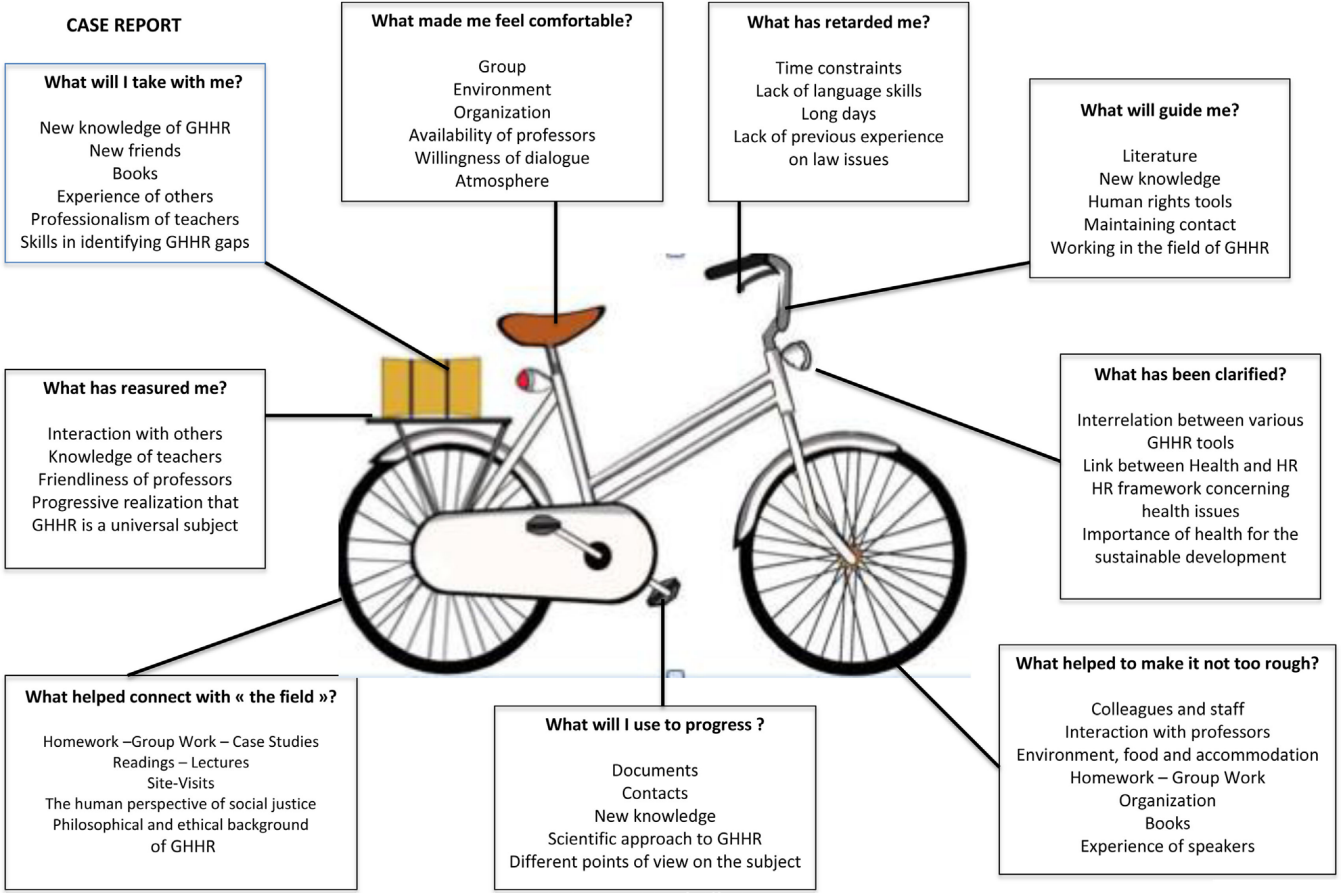
Global evaluation from successive cohorts: “What is the contribution of the program to me personally?”

Several evaluation procedures were adopted. First, we adopted a fairly recently developed evaluation tool for teaching programs “Le petit vélo” (36), an open-ended questionnaire whose objective is to make the evaluation process more fun while allowing the collection of precise information. Figure 1 shows pooled data of most frequent answers to a series of questions the students of the successive cohorts were asked in a written form at the end of the program. Interactions with fellow students, teaching staff, and experts with field experience were most frequently positively mentioned: these interactions were facilitated through group work, journal clubs, and informal discussions. For many students, the issues discussed were completely new and they were impressed/overwhelmed by their public health importance.

Second, the students’ satisfaction was monitored through a SWOT technique (37). It is a widely used evaluation technique in education able to provide information that should be taken into account in order to improve the program/curriculum. The variables of interest are grouped in four categories, usually identified with the letters S, W, O, and T (successes, weaknesses, opportunities, threats). In the present context, the technique was used at the end of the program during a residential session where each participant had the opportunity to identify four elements in each category. The data was then pooled and analyzed. The most frequently mentioned points, ranked from 1 to 4, are presented in Table 5. Quite clearly, the site visits at IOs, such as WHO, ILO, or ICRC, allowing the students to discover the organization, their projects, and their commitment in the field of health and human rights were most widely appreciated. The opportunity of networking with students from various educational and cultural backgrounds was also very positively mentioned.

**Table 5:** Students’ satisfaction via SWOT technique: most frequently mentioned points ranked from 1 to 4.

Successes	Opportunities
Site visits Quality of organization, logistics, and material Students from various backgrounds (country/study field) Quality of lectures by academics and experts with field experience	Possibility of networking among students Possibility of networking with non-governmental organizations and international organizations Acquisition of new knowledge on various topics Intercultural experience

**WEAKNESSES**	**THREATS**

Little time for discussions Too many topics Long hours Limited feedback of group work and case studies	Time constraints Heavy workload Lack of language skills High fees

Third, the learning outcomes were evaluated through several pass/fail examinations, including an individual journal article presentation, a group work assignment with a powerpoint presentation to the whole class, and an individual written essay based on situation analysis or case studies. Two to three teachers of the coordination staff were responsible of the pass/fail appreciations. The attendance of class, the participation at the group works and the site visits, and the completion of the certifying assignments make up for three ECTS credits.

The possible impact of the course on the career of the participants was not investigated. However, a survey is planned in order to assess this dimension, a survey similar to the one done among alumni of the Master of Advanced Studies in Public Health of the University of Geneva (38).

## Discussion

Summer schools are an increasingly popular kind of education that is organized by universities during the summer break. The offer all over the world is very important. Some summer schools are full-time intense courses focusing on specific subjects/topics/methodologies. Others are less intensive and also offer a cultural program and language courses. The duration of a summer school varies from anything from 2 days up to several weeks. Participation at a summer school yields multiple benefits to the participants, as well personally as socially and academically (39). Specific e-portals exist allowing students to access to several hundreds of summer courses. One of them lists 1,654 summer school programs worldwide adding a short description to each, yet not pretending to be exhaustive (40). With a “human rights filter,” the portal yields 53 courses; when checked with a “health and human rights filter” it indicates 10 courses, ranging from 5 days to 8 weeks and mostly located at European Universities, such as Utrecht, Groningen, Rotterdam, Aahrus, Rome, and Giessen.

According to the Office of the UN High Commissioner on Human Rights, “Human rights education constitutes an essential contribution to the long-term prevention of human rights abuses and represents an important investment in the endeavor to achieve a just society in which all human rights of all persons are valued and respected” (41). Regarding “health and human rights,” there are several issues to consider: first, health personals as citizens “must uphold truth and human rights” (42); second, health personals as professionals are potentially in charge of patients at risk of discrimination and stigmatization because of their health problem (43), which implies that they have obligations in protecting the basic rights of their patients; third, health professionals may become “complicit in serious human rights abuses” (44). It therefore appears fundamental to train professionals in the field of health and human rights, as has been recommended in the literature (45). A crucial question remains open: are such training programs transformative, i.e., empowering trainees in such a way they produce changes in their working environments and their communities as suggested by some authors (46, 47)?

The “Global Health and Human Rights Summer-School” of the University of Geneva is in phase with the 1994 UN resolution proclaiming a Decade of Human Rights Education and the UN World Program for Human Rights Education launched in 2005 (48, 49). More recently, the importance of education and training in human rights has further been stressed by a high-level panel discussion of the Human Rights Council on the implementation of the UN Declaration on Human Rights Education and Training (50).

The “Global Health and Human Rights Summer-School” of the University of Geneva has heavily benefited from the inputs and support of experts from IOs and NGOs active in the field of Health and Human Rights and located in Geneva, such as WHO, ILO, ICRC, and MSF as well as from academics with expertise in law, human rights legislation, and international studies.

Several educational approaches were adopted with special focus on active learning processes as recommended by adult learning theorists/promoters (51, 52), which allowed exchange of experiences and networking among students, two points very positively evaluated, and active networks do contribute to quality of public health programs (53). Case studies and group work on specific topics also allowed in depth work by students and fostered brainstorming and vivid discussions, as has been reported in the literature (54). Unquestionably, the site visits at the IOs were most widely appreciated by the students: those visits allowed the students to get acquainted with projects and programs in the field of health and human rights of these IOs; they also allowed exchanges with professionals having been confronted to a wide range of health and human rights challenges during their carrier: this in turn enabled students to link theory to practice, a highly recommended process in consolidating newly acquired knowledge (55).

A major challenge for most of the students has been the workload due to the requirements of the program and the related time constraints, which was considered a threat; yet, one could argue that managing a heavy workload and time constraints is a skill useful to any professional (56).

We adopted several evaluation tools in order to better identify strengths and weaknesses of the program: one is the SWOT technique which has been shown to yield interesting information on educational programs (37); one is the “Petit Vélo” tool (36) which specifically gives the students the opportunity to reflect upon nine topics that either relate to the dynamics of the course (e.g., what has reassured me?), to its content (e.g., what has been clarified?), or to its future impact (e.g., what will I use to progress).

The program itself faces several challenges, mainly:

– funding difficulties: the University expects the summer school programs to be financially self-sustaining, which is hardly possible based on the sole tuition fees; thus, external funding must be sought, which at time can be facetious;
– lack of institutional recognition: despite the fact that the summer school project is high on the university agenda, each program is embedded in a specific faculty/school (the Medical School in our case) whose priorities are mostly high-level research and pre-graduate education to regular students and not summer school programs that add to the traditional activities of the faculty/school; the corollary of this is a disregard of those activities for professional promotion;
– extra workload for the teaching staff in an already loaded calendar.

The main lesson is that educational recommendations of the UN Manual on Human Rights Training Methodology insisting on an active pedagogy focused on student interests and needs complemented by field visits (in this case to the Geneva-based IOs) have been extremely useful and have been very helpful, certainly contributing to the satisfaction of the students, their commitment, and their enthusiasm.

## Conclusion

The *Global Health and Human Rights Summer-School* of the University of Geneva allowed its participants, coming from all over the world, to identify challenges of the interlinked fields of health and human rights, reflect upon their underlying causes and imagine possible solutions.

Given the success met, we are considering offering to students interested in deepening their understanding of the health and human rights problematic the opportunity to pursue their studies with a distance learning program allowing them to get a CAS of the University of Geneva. A first experience was positive.

We share the present experience in the spirit as expressed by Ewert et al. (57): “*information and experiences [should] be shared with others in the health and human rights educational community*.” We also hope that sharing our experience might be an incentive for passionate educators around the world to develop similar programs.

## Author Contributions

The two authors designed the program; EM collected and analyzed the data; PC drafted the paper. Both authors read and validated the final text.

## Conflict of Interest Statement

The authors declare that the research was conducted in the absence of any commercial or financial relationships that could be construed as a potential conflict of interest.
